# The Registration of Nurses Bill

**Published:** 1919-11-22

**Authors:** 


### NURSES' REGISTRATION (No. 2) BILL.

### PRESENTED BY DR. ADDISON.

ARRANGEMENT OF CLAUSES.

1. Establishment and constitution of General Nursing Council.
2. Register of nurses.
3. Rules.
4. Staff and expenses.
5. Fees.
6. Admission to Register of persons trained outside United Kingdom.
7. Appeal against removal from Register, and against refusal to approve institution.
8. Penalties for unlawful assumption of title of registered nurse and for falsification of Register.
9. Extent and short title.
SCHEDULE.

### THE REGISTRATION OF NURSES BILL.

BE it enacted by the King's most Excellent Majesty, by and with the advice and consent of the Lords Spiritual and Temporal, and Commons, in this present Parliament assembled, and by the authority of the same, as follows :—

ESTABLISHMENT AND CONSTITUTION OF GENERAL NURSING COUNCIL.

1.—(1) For the purposes of this Act there shall be established a General Nursing Council (in this Act referred to as "the Council"), which shall be a body corporate by that name with perpetual succession and a common seal with power to acquire and hold land without licence in mortmain.

(2) The Council shall be constituted in accordance with the provisions contained in the Schedule to this Act.

(3) The seal of the Council shall be authenticated in the prescribed manner and any document purporting to be sealed with the said seal so authenticated shall be receivable in evidence of the particulars stated in that document.

REGISTER OF NURSES.

2.—(1) It shall be the duty of the Council to form and keep a register of nurses (in this Act referred to as "the register") subject to and in accordance with the provisions of this Act.

(2) The register shall consist of the following parts :—

(*a*) a general part containing the names of all nurses other than nurses to be included in some other part of the register;

(*b*) a supplementary part containing the names of male nurses;

(*c*) a supplementary part containing the names of mental nurses;

(*d*) a supplementary part containing the names of nurses trained in the nursing of sick children;

(*e*) any other prescribed part.

(3) A certificate under the seal of the Council duly authenticated in the prescribed manner stating that any person is, or was at any date, or is not, or was not at any date, duly registered under this Act shall be conclusive evidence in all courts of law of the fact stated in the certificate.

RULES.

3.—(1) The Council shall make rules for the following purposes :—

(*a*) for regulating the formation, maintenance and publication of the register;

(*b*) for regulating the conditions of admission to the register;

(*c*) for regulating the conduct of any examinations which may be prescribed as a condition of admission to the register, and any matters ancillary to or connected with any such examinations;

(*d*) for prescribing the causes for which, the conditions under which, and the manner in which nurses may be removed from the register, the procedure for the restoration to the register of nurses who have been removed therefrom, and the fee to be payable on such restoration;

(*e*) for regulating the summoning of meetings of the Council and the proceedings (including quorum) of the Council;

(*f*) generally for making provision with respect to any matters with respect to which the Council think that provision should be made for the purpose of carrying this Act into effect (including provision with respect to the issue of certificates to nurses registered under this Act and with respect to the uniform or badge which may be worn by nurses so registered), and for prescribing anything which under this Act is to be prescribed.

(2) Rules under this section shall contain provisions—

(*a*) requiring as a condition of the admission of any person to the register that that person shall have undergone the prescribed training, and shall possess the prescribed experience, in the nursing of the sick; and

(*b*) requiring that the prescribed training shall be training either in an institution approved by the Council in that behalf or in the service of the Admiralty, the Army Council, or the Air Council; and

(*c*) enabling persons who, within a period of two years after the date on which the rules to be made under the provisions of this paragraph first come into operation, make an application in that behalf (in this Act referred to as "an existing nurse's application"), to be admitted to the register on producing evidence to the satisfaction of the Council that they are of good character, are of the prescribed age, are persons who were for at least three years before the first day of November, nineteen hundred and nineteen, *bonâ fide* engaged in practice as nurses in attendance on the sick under conditions which appear to the Council to be satisfactory for the purposes of this provision and have adequate knowledge and experience of the nursing of the sick.

(3) Rules made under this section shall not come into operation unless and until they are approved by the Minister of Health.

(4) Every rule made under this section shall be laid before each House of Parliament forthwith, and if an address is presented to His Majesty by either House of Parliament within the next subsequent twenty-one days on which that House has sat next after any such rule is laid before it praying that the rule may be annulled, His Majesty in Council may annul the rule and it shall thenceforth be void, but without prejudice to the validity of anything previously done thereunder.

STAFF AND EXPENSES.

4.—(1) The Council may, with the previous sanction of the Minister of Health, appoint a person to act as registrar of the Council, and may, subject to the consent of the Minister as to numbers, employ such other officers as the Council consider necessary.

**Nurses' Registration (No. 2) Bill**—(*continued*).

(2) There shall be paid to the registrar and the officers of the Council such salaries or remuneration as the Council with the approval of the Minister of Health may from time to time determine.

(3) Any expenses incurred by the Council in carrying this Act into effect, including expenses in connection with examinations or prosecutions under this Act and, subject as hereinafter provided, the travelling expenses of and sums paid on account of subsistence allowance to members of the Council, shall be defrayed out of the sums received by the Council by way of fees under this Act:

Provided that the amount to be allowed to members of the Council in respect of travelling expenses and subsistence allowance shall be calculated in accordance with directions to be given by the Minister of Health.

(4) The accounts of the Council shall be audited in such manner, and by such person, as the Minister of Health may from time to time direct, and copies of the accounts, and of any report made on the accounts, shall be transmitted by the Council to such persons as the Minister may direct.

Fees.

5.—(1) There shall be paid to the Council in respect of every application to be examined or to be registered under this Act, and in respect of the retention in any year of the name of any person on the register, such fees respectively as the Council may, with the approval of the Minister of Health, from time to time determine:

Provided that—

(*a*) in the case of an existing nurse's application the amount of the fee payable on the application shall be such sum, not exceeding one guinea, as the Council, with such approval as aforesaid, may determine; and

(*b*) the amount of the fee payable in respect of the retention in any year of the name of any person on the register shall not exceed two shillings and sixpence.

(2) The Council may charge for any certificate or other document issued, or in respect of any services performed, by them, such fees as may be prescribed.

Admission to Register of Persons Trained Outside United Kingdom.

6.—(1) Any person who proves to the satisfaction of the Council that he has been registered as a nurse in any part of His Majesty's dominions outside the United Kingdom, being a part of those dominions to which this section applies, shall be entitled, on making an application in the prescribed manner and paying such fee, not being greater than the fee payable on ordinary applications for registration under this Act, as the Council may demand, to be registered under this Act.

(2) This section applies to any part of his Majesty's Dominions as respects which the Council are satisfied—

(*a*) that there is in force therein an enactment, or a provision of any kind having the force of law, providing for the registration of nurses under some public authority;

(*b*) that persons registered under this Act are admitted to the register established under the said enactment or provision on terms not less favourable than those contained in sub-section (1) of this section; and

(*c*) that the standard of training and examination required for admission to the register of nurses established under the said enactment or provision is not lower than the standard of training and examination required under this Act.

(3) In the event of provision being hereafter made for the establishment of a register of nurses in Scotland or Ireland the Council shall make rules under this Act enabling persons registered as nurses in Scotland or Ireland, as the case may be, to obtain admission to the register of nurses established under this Act.

Appeal Against Removal from Register, and Against Refusal to Approve Institution.

7.—(1) Any person aggrieved by the removal of his name from the register may, within three months after the date on which notice is given to him by the Council that his name has been so removed, appeal against the removal in manner provided by rules of court to the High Court, and on any such appeal the High Court may give such directions in the matter as it thinks proper, including directions as to the costs of the appeal, and the order of the High Court shall be final and conclusive and not subject to an appeal to any other court.

(2) Any person aggrieved by the refusal of the Council to approve any institution for the purpose of the rules under this Act relating to training may appeal against the refusal to the Minister of Health, and the Minister, after considering the matter, shall give such directions therein as he thinks proper, and the Council shall comply with any directions so given.

Penalties for Unlawful Assumption of Title of Registered Nurse and for Falsification of Register.

8.—(1) If any person, not being a person duly registered under this Act, at any time after the expiration of three months from the date on which the Minister of Health gives public notice that a register of nurses has been compiled under this Act, takes or uses the name or title of registered nurse, either alone or in combination with any other words or letters, or any name, title, addition, description, uniform, or badge, implying that he is registered under this Act or is recognised by law as a registered nurse, or with intent to deceive makes use of any certificate of registration as a nurse issued under this Act to him or any other person, he shall be liable on summary conviction to a fine not exceeding, in the case of a first offence, ten pounds, and in the case of a second or any subsequent offence fifty pounds.

(2) If any person wilfully makes, or causes to be made, any falsification of any matter relating to the register, he shall be guilty of a misdemeanour, and shall, on conviction thereof, be liable to a fine not exceeding one hundred pounds.

Extent and Short Title.

9.—(1) This Act shall not extend to Scotland or Ireland.

(2) This Act may be cited as the Nurses' Registration (No. 2) Act, 1919.

---

### NURSES' REGISTRATION (No. 2) BILL. SCHEDULE.

Constitution of Council.

1. The Council shall consist of twenty-five members.

2. On its first constitution the Council shall be composed of the following persons, namely:

Two persons, who shall not be registered medical practitioners, or nurses, or persons concerned with the regular direction or provision of the services of nurses, appointed by the Privy Council.

Two persons appointed by the Board of Education.

Five persons appointed by the Minister of Health, after consultation with persons and bodies having special knowledge and experience of training schools for nurses, of the work of matrons of hospitals, of general and special

**Nurses' Registration (No. 2) Bill**—(*continued*).

nursing services, and of general and special medical practice.

Sixteen persons, who are or have at some time been nurses actually engaged in rendering services in direct connection with the nursing of the sick, appointed by the Minister of Health after consultation with the Central Committee for the State Registration of Nurses, the College of Nursing, the Royal British Nurses' Association, and such other associations or organised bodies of nurses or matrons as represent to the Minister that they desire to be consulted in the matter.

3. The first members of the Council shall hold office for such term, not exceeding three years from the commencement of this Act, as the Minister of Health may determine.

4. After the expiration of the term of office of the first members of the Council, the Council shall be composed of nine persons appointed respectively by the Privy Council, the Board of Education, and the Minister of Health as aforesaid, and of sixteen persons, being persons registered as nurses under this Act, elected in accordance with the prescribed scheme and in the prescribed manner by the persons so registered at the date of election.

5. Any members of the Council other than the first members thereof shall hold office for a term of five years.

6. If the place of a member of the Council becomes vacant before the expiration of his term of office, whether by death, resignation, or otherwise, the vacancy shall be filled by appointment by the body or persons by whom the member was appointed, or if the vacating member was an elected member by the Council.

The Council in co-opting a member under the foregoing provision shall, so far as practicable, select a person, being a person registered as a nurse under this Act, who is representative of the same interests as those represented by the vacating member.

Any person appointed or elected to fill a casual vacancy shall hold office only so long as the member in whose stead he is appointed or elected would have held office.

7. Any member ceasing to be a member of the Council shall be eligible for re-appointment or re-election.

8. The powers of the Council may be exercised notwithstanding any vacancy in their number.

